# Machine learning methods in chemoinformatics

**DOI:** 10.1002/wcms.1183

**Published:** 2014-02-24

**Authors:** John B O Mitchell

**Affiliations:** School of Chemistry, University of St AndrewsSt Andrews, UK

## Abstract

Machine learning algorithms are generally developed in computer science or adjacent disciplines and find their way into chemical modeling by a process of diffusion. Though particular machine learning methods are popular in chemoinformatics and quantitative structure–activity relationships (QSAR), many others exist in the technical literature. This discussion is methods-based and focused on some algorithms that chemoinformatics researchers frequently use. It makes no claim to be exhaustive. We concentrate on methods for supervised learning, predicting the unknown property values of a test set of instances, usually molecules, based on the known values for a training set. Particularly relevant approaches include Artificial Neural Networks, Random Forest, Support Vector Machine, k-Nearest Neighbors and naïve Bayes classifiers. *WIREs Comput Mol Sci* 2014, 4:468–481.

**How to cite this article:** *WIREs Comput Mol Sci* 2014, 4:468–481. doi:10.1002/wcms.1183

## INTRODUCTION

The field known as chemoinformatics, or sometimes cheminformatics, can be considered as that part of computational chemistry whose models are *not* based on reproducing the real physics and chemistry by which the world works at the molecular scale. Unlike quantum chemistry or molecular simulation, which are designed to model physical reality, chemoinformatics is intended simply to produce useful models that can predict chemical and biological properties of compounds given the two-dimensional (or sometimes three, see Box 1 chemical structure of a molecule.

**REPRESENTING MOLECULES: TWO OR THREE-DIMENSIONAL?**

In chemoinformatics, the researcher is presented with a fundamental dilemma—should the molecules be described with two- or three-dimensional representations? A two-dimensional representation is essentially a molecular graph with the atoms as nodes and the bonds as edges. Onto this may be added extra information, such as bond orders, and the stereochemistry about double bonds and at chiral centers. Such a representation of chemical structure is essentially a digitized form of the structural diagrams familiar to chemists, and lacks the explicit spatial coordinates of the atoms.

An alternative approach is to generate a three-dimensional structure. This can be done from the molecular graph or connection table using a program such as CORINA,^7^,^8^ from a crystal structure, or from a quantum chemical calculation. Although a three-dimensional structure carries additional information, the difficulty is that molecules generally exist as an equilibrium between multiple conformers. Even if our structure correctly represents the lowest energy, and hence most abundant, conformer, alternative conformations may be critical for biological functions such as protein binding. Nonetheless, using three-dimensional structure opens up possibilities like scaffold hopping in drug design, where molecules with diverse two-dimensional structures but similar three-dimensional shapes may bind the same target. Sheridan and Kearsley's review^9^ and an article by the Ritchie group^10^ are two of the numerous papers discussing the relative merits of two and three-dimensional molecular representations.

The history of chemoinformatics began with local models, typically for quantitative structure–activity relationships (QSAR) or quantitative structure–property relationships (QSPR). Popular versions of this history usually begin with Hammett or Hansch,^1^,^2^ though Borman has followed the trail of QSAR back into the 19th century.^3^ Early models were generally based on linear, and later multilinear, regression. These were typically built using only a very few features, and were valid only for a small series of closely related compounds. Interestingly, machine learning and pattern recognition methods have an association with chemistry going back more than four decades, with methods like the linear learning machine being applied to problems such as the interpretation of spectroscopic data, as discussed in an early review by Kowalski.^4^

In contrast to the very small applicability domains of early QSAR studies, much recent work has concentrated on global models, by which we mean models trained on and hence valid for a wide range of organic or drug-like compounds. A number of factors, most notably the availability of data for molecules spanning a much wider chemical space, the use of a large and diverse selection of descriptors, and the development of sophisticated nonlinear machine-learning algorithms have increased the use of such global models in recent years.

## CHEMOINFORMATICS

### From Molecules to Features to Properties

**Figure 1:**
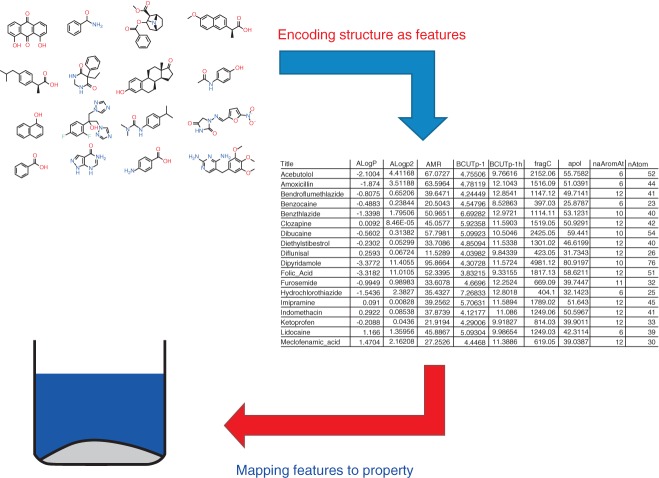
We can conceive of chemoinformatics as a two-part problem: encoding chemical structure as features, and mapping the features to the output property. The second of these is most often the province of machine learning.

Although to some extent a postrationalization, it is helpful to consider chemoinformatics model building as a two-part process.^5^ Firstly a molecular structure, typically represented as a molecular graph or connection table, is converted into a vector of features (which are also known as descriptors and represented generically by the symbol *x*). This first stage may sometimes also use three-dimensional information (see Box 1), and can be referred to as the *encoding*. Numerous recipes, some freely available and many commercial, exist for encoding a compound as a feature vector.^6^ The second part of building a machine-learning model for chemoinformatics is the *mapping* (using Lusci et al.'s terminology^4^). This involves empirically discovering a function that maps between the feature vectors and the property of interest, represented by the symbol *y*. It is this mapping that is most often learnt by the machine-learning algorithm. The two-part process is illustrated in Figure 1.

#### Feature Selection

The descriptors chosen to represent the molecules have no *a priori* reason either all to be relevant for describing the property to be predicted or all to be independent of one another. Some machine-learning algorithms are robust against the inclusion of irrelevant or of mutually correlated features, others less so. It is fairly common, as part of the training phase of the algorithm, to choose a subset of the original features that are helpful for building a predictive model and not strongly correlated with one another.^11^–^13^ An alternative approach is principal component analysis (PCA),^14^ a statistical procedure that transforms mutually correlated variables, which should first be scaled, into mutually uncorrelated combinations called principal components. The process maximizes the variance of the first principal component; the components are ordered from first downwards by decreasing variance. PCA provides a way of explaining most of the variance in the output variable with a small number of orthogonal components. Each principal component is a linear combination of the original features, so although the linear combinations do not have such clear meanings as the original features, the user can at least see which features are contributing to the model.

#### Similar Property Principle

Two compounds that are ‘similar’ to one another will have feature vectors that, when considered as position vectors in the chemical space spanned by the descriptors, are close to each other. If the mapping function varies reasonably slowly and smoothly across chemical space, then we expect similar molecules to have similar values of the relevant chemical (or biological) property. This is the basis of the similar property principle: ‘Similar molecules have similar properties’. While this may be considered a central principle of chemoinformatics, it is far from universally valid. In the case of ‘activity cliffs’ for instance, the mapping function varies dramatically over a small distance in chemical space, corresponding perhaps to a change of one functional group which might prevent a ligand from binding effectively to a protein, and apparently similar molecules can have very different bioactivities.^15^

### What Properties Can We Model?

For many properties, especially those of isolated molecules, chemoinformatics and machine learning would be a poor choice of methodology. If we want to calculate dipole moments, polarizabilities or vibrational frequencies, we would be better off using quantum chemistry. On the other hand, where a complex biological or condensed phase system cannot easily be directly modeled by physics-based methods, then chemoinformatics becomes a sensible option. Given the extent to which the development of chemoinformatics has been intertwined with drug discovery and the pharmaceutical industry, it is hardly surprising that bioactivity and ADMET (Absorption, Distribution, Metabolism, Excretion and Toxicity) properties rank high on the list of those addressed by informatics approaches. There are also numerous physicochemical properties that are hard to obtain from theoretical chemical methods such as density functional theory or molecular dynamics, and hence are often modeled by chemoinformatics. Amongst such properties, aqueous solubility and logP (the logarithm of the octanol:water partition coefficient) are directly relevant to drug discovery, and melting point is indirectly so, due to its correlation with solubility.^16^ Properties such as solubility^17^,^18^ and sublimation energy^19^–^21^ are potentially amenable to modeling by either chemoinformatics or theoretical chemistry approaches.

## MACHINE-LEARNING METHODS

### Artificial Neural Networks

An artificial neural network (ANN), often simply called a neural network where confusion with biology is unlikely, is a mathematical model used for pattern recognition and machine learning. The network's architecture is based on connected neurons in an input layer, a hidden layer or layers, and an output layer. In a typical design, each connection between neurons carries a weight. The weights are varied during the training phase as the network learns how to connect input and output data, before being tested on unseen instances. While the ANN is inspired by the structure and function of the human brain, it is massively simpler in design and in no way simulates higher brain function. In fact, a typical ANN will be smaller than the minimal 302 neuron brain of the nematode *Caenorhabditis elegans*.^22^

ANNs have been used for a wide range of chemoinformatics applications. Amongst studies seeking to predict bioactivity are Li et al.'s work on estrogen receptor agonists,^23^ and So et al.'s study of steroids,^24^ while neural networks were amongst a number of methods used by Briem et al. to identify possible kinase inhibitors.^25^ As with other machine-learning methods, ANNs are often used to predict toxicological, pharmacological, and physicochemical properties, such as hERG blockade,^26^ aquatic toxicity,^27^ drug clearance,^28^ pKa,^29^ melting point,^30^,^31^ and solubility.^17^,^32^,^33^ However, ANNs suffer from vulnerability to overfitting, with a danger of learning the noise as well as the signal from the training set and hence being less able to generalize to the unseen test data.^34^ Addressing this requires careful study design, so that the training process can be stopped close to the optimal time.

#### Deep Learning

Deep learning, a concept closely associated with ANNs, is in principle the learning of layered concepts. Thus, a model could describe higher and lower-level concepts at different layers of its structure. Lusci et al.^5^ use a multilayer ANN, which they describe as a deep learning technique, to go from molecular graphs to a set of descriptors and then, *via* a suitable output function, to predict aqueous solubility, as shown in Figure 1. Overall, they are able to generate good predictions of solubility, competitive with other sophisticated machine-learning methods. Their model is in fact an ensemble of 20 different ANNs, each with slightly different architectures.

#### The Wisdom of Crowds and Ensembles of Predictors

The *wisdom of crowds* is a well-known expression of the benefit of utilizing multiple independent predictors.^35^ For example, a fairground competition might involve members of the public guessing the weight of a cow. No doubt, some guesses would be far too high and others much too low. However, the *wisdom of crowds* concept holds that the ensemble of estimates is capable of making an accurate prediction, as observed by Galton more than a century ago.^36^ In order to avoid the excessive effect of one or two absurd guesses on the mean, it is preferable to use the median of the estimates as the best prediction in the context of a public guessing game.

This idea was exploited by Bhat et al.,^29^ who used an ensemble of neural networks to predict the melting points of organic compounds. Each network has a different, randomly assigned, set of initial weights. The authors found a significant improvement in prediction accuracy as a result of using the ensemble approach, with the ensemble prediction being better not just than that of a typical single network, but better than the best performing single ANN. They achieved substantial improvement upon adding the first few additional ANNs, but there was little further effect in going beyond a few tens of networks. The authors chose to use 50 ANNs in their final model, though the rapid training time (around 9 seconds per network) meant that they could have afforded more if required. While Bhat et al. adapted an ANN approach to benefit from using an ensemble of predictors, we will see that the use of multiple independent models is also fundamental to Random Forest (RF). The key to benefitting from the *wisdom of crowds* is to design an algorithm that can produce multiple *independent* predictors, even though their predictions must be based on essentially the same pool of data. Interestingly, the idea of combining predictors of different kinds into an ensemble has been much less explored in chemoinformatics than in postdock scoring, where consensus scores constitute a well-established method.^37^

### Random Forest

**Figure 2:**
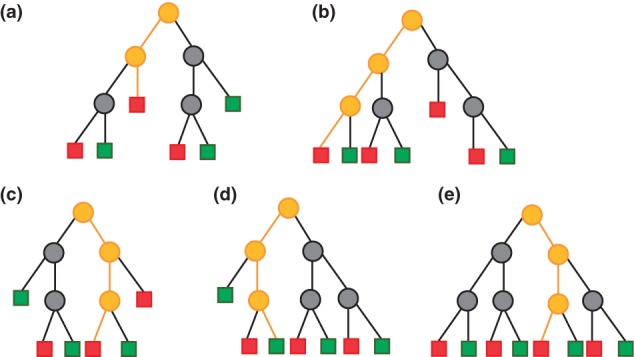
Five illustrative decision trees forming a (very small) Random Forest for classification. The terminal leaf nodes are shown as squares and colored red or green according to class. The path taken through each tree by a query instance is shown in orange. Trees A, B, C, and E predict that the instance belongs to the red class, tree D dissenting, so that the Random Forest will assign it to the red class by a 4–1 majority vote.

RF^38^,^39^ is a technique for classification based on an ensemble, or forest, of decision trees. The large number of independent trees allows RF to benefit from the *wisdom of crowds* effect. The trees are built using training data consisting of multiple features for each of a training set of objects. As we are discussing chemical applications, we will assume that these objects or instances are molecules. Each tree is generated by stochastic recursive partitioning and is randomized in two ways. Firstly, the tree is randomized by allowing it to use, at each node, only a stochastically chosen subset of the features. As the training instances progress through the tree, they are partitioned into increasingly homogeneous groups, so that each terminal node of the decision tree is associated with a group of molecules with similar values of the property to be predicted. Each split within a tree is created based on the best partitioning that is possible according to the Gini criterion,^40^ using any single-valued attribute from a randomly chosen subset of descriptors. The number of descriptors in this random subset is the parameter *mtry*, and the subset is freshly chosen for each node. The tree building continues until all training instances have been assigned to a terminal leaf node, see Figure 2.

Secondly, each tree is randomized by basing it on a bootstrap sample of the training data. From a pool consisting of *N* distinct objects, a sample of *N* objects is chosen with replacement, so that each object may be chosen zero, one, two, or occasionally more times. The probability of a given molecule not being chosen for a given tree's bootstrap sample is (1 – 1/*N*)*^N^*, which tends to a limit of 1/e, or approximately 0.37, as *N* becomes large. Thus, for each tree, approximately 37% of the training set molecules do not appear in that tree's bootstrap sample, and constitute the so-called out-of-bag data; conversely, every molecule is out-of-bag for about 37% of the trees. The out-of-bag sample can be used as an internal validation set for each tree; the performance on the different out-of-bag samples of each tree provides a fair test of the predictivity of the RF.

The RF consists of *ntree* stochastically different trees, each built from its own bootstrap sample of the training data. The trained forest is then used to predict unseen test data. In the case of predicting a binary or a multiclass categorical variable, the classifications are determined by majority vote amongst the trees. The proportion of votes cast for a class may provide an indication of the probability of a label being correctly assigned, or of confidence in a prediction, but this should be considered an informal estimate only.^41^ Similarly, a RF of regression trees can be used for predicting numerical quantities, with the predictions from the different trees being averaged. For classification, the default value of *mtry* recommended by Svetnik et al.^39^ is the square root of the total number of descriptors; for regression, they advise using *mtry* equal to one third of the number of descriptors. If *mtry* were increased to equal the full number of descriptors, RF would become equivalent to another machine-learning method, the bootstrap aggregating technique known as bagging. Alternatively, *mtry* could be treated as an optimizable parameter. A typical default value of *ntree* is 500, though there is a case for using an odd number of trees in binary prediction to avoid ties (which would be resolved randomly). Often, RF calculations are relatively cheap and a larger number of trees could be afforded; however, the improvement in prediction accuracy with additional trees is small. RF is generally considered relatively robust against overfitting. Svetnik et al. demonstrate that, unlike ANN, the test set error of RF does not increase but converges to a limiting asymptotic value as the training error is reduced toward zero, one of many interesting observations contained in an excellent paper that describes RF in full detail.^39^

RF has proven a very successful method in chemoinformatics and has been used in many different contexts. These include prediction of athletic performance enhancement,^42^ QSAR,^43^–^45^ mutagenicity,^46^ phospholipidosis,^47^ hERG blockade,^48^ and skin sensitization.^49^ Applications in physicochemical properties include discovery of new crystalline solvates^50^ and solubility,^51^ the prediction of which has also been systematically compared with those of melting point and logP.^52^ RF has also found applications in the area of postdock scoring functions and predicting protein–ligand binding affinity.^53^–^56^

### Support Vector Machine

Support Vector Machine (SVM)^57^ maps the data into a high-dimensional space, using a kernel function that is typically nonlinear. The SVM seeks to find an optimal separation between two classes, such that each in their entirety lie on opposite sides of a separating hyperplane. This is achieved by maximizing the margin between the closest points, known as support vectors, and the hyperplane. SVM can be adapted to either multiclass classification^58^,^59^ or to regression. SVMs are one of the most popular machine-learning methods in chemoinformatics. Uses in bioactivity prediction include drug repurposing,^60^,^61^ kinase inhibition,^25^ estrogen receptor agonists,^23^ and opioid activity.^62^ The SVM is often used to predict toxicity-related properties such as hERG blockade,^47^,^63^,^64^ mutagenic toxicity,^65^ toxicity classification,^66^ and phospholipidosis.^47^,^67^ Applications in physicochemical property prediction include solubility,^33^,^52^,^68^ pKa,^29^ logP, and melting point.^52^ The interested reader is referred to Noble's instructive article for further discussion of SVMs.^57^

### k-Nearest Neighbors

**Figure 3:**
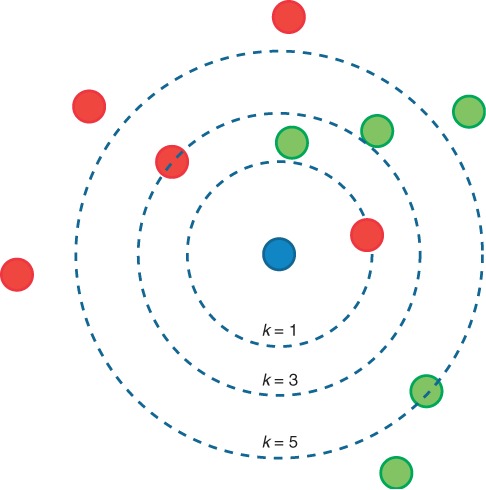
Illustration of a kNN classification model. For *k* = 1, the model will classify the blue query instance as a member of the red class; for *k* = 3, it will again be assigned to the red class, this time by a 2–1 vote; however, since the fourth and fifth nearest neighbors are both green, a *k* = 5 model would classify it as part of the green class by a 3–2 majority.

The *k*-nearest neighbors (kNN) algorithm is one of the simplest machine-learning methods to understand and explain, the principle being that an instance is classified by a majority vote of its neighbors, see Figure 3. Each test instance is predicted to belong to the class most commonly found amongst its k closest neighbors, where *k* is a positive integer. Most often, *k* is chosen to be small; if *k* = 1, the instance is simply assigned to the same class as its nearest neighbor in a feature space. The instances, which in chemical applications are typically molecules, are described as position vectors in the feature space, which is usually of high dimensionality. It is helpful to scale the features so that distances measured in different directions in the space are comparable. Neighbors are identified on the basis of distance in the feature space. This is usually taken to be the Euclidean distance, though other metrics such as the Jaccard distance could be used. In binary classification problems, it is helpful to choose *k* to be an odd number as this reduces the risk of tied votes, though depending on the granularity of the space multiple neighbors may share the same distance. Once the labeled instances and their positions in the feature space are available, no explicit training phase is required. Because of this, kNN is considered a ‘lazy learning’ algorithm.

The same method can be used for regression. This is most simply achieved by assigning the property for the test instance to take the mean value calculated from its k closest neighbors. However, it can be helpful to weight the contributions of the neighbors, the closest neighbor contributing most to the average and the *k*th neighbor contributing least; a procedure for doing this was published by Nigsch et al.^69^ This effectively smooths the transition between neighboring instances being counted and non-neighboring instances being ignored.

The kNN algorithm is sensitive to the local structure of the data. Thus it is ideal for calculating properties with strong locality, as is the case with protein function prediction.^70^ If a single neighbor with, say, 90% identity to the test sequence is found, it is highly likely that the functional label can be safely transferred to the query sequence; a dozen neighbors each with 25% identity would be much less useful. Despite its locality, kNN can still give global coverage, so long as the instances are distributed throughout the feature space. In principle, the distance to the neighbors, or the proportions of neighbors having a given label, could be used to measure prediction confidence, though this is rarely done in practice.

Many studies use some kind of internal validation to optimize the value of *k*, with the optimum value dependent upon the dataset at hand. kNN has been used in bioactivity studies of anticonvulsants and dopamine D1 agonists^71^ of kinase inhibition,^25^ cannabinoid psychoactivity,^72^ steroids, anti-inflammatories and anti-cancer drugs,^73^ athletic performance enhancement,^42^ and estrogen receptor agonists.^23^ Studies of toxicological and pharmacological relevance have looked at drug clearance,^28^ mutagenic potency,^74^ and percutaneous drug absorption.^75^ kNN has been used to predict the odor characteristics of compounds.^76^,^77^ Studies using kNN to investigate physicochemical properties have considered melting point,^52^,^69^ boiling point,^78^ logP,^52^,^78^ aqueous solubility,^52^,^79^ and the analysis of mixtures.^80^

### Naïve Bayes

The naïve Bayes classifier provides a rather different kind of algorithm, one based on estimating the probabilities of class membership. Application of Bayes' theorem, together with the assumption that the features *x*_i_ are conditionally independent of one another given the output class *y*, leads to the formula 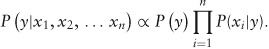


The decision rule is to assign a test instance to the class with the highest estimated probability. Undoubtedly, the assumption of conditional independence of the features is not strictly valid. Nonetheless, naïve Bayes often performs well enough to be competitive with other machine-learning methods, and has the advantage of conceptual simplicity compared to most. As early as 1974, Cramer et al. published a method of computing the conditional probability of a molecule being bioactive given the fragments it contained.^81^ Naïve Bayes classifiers are now frequently used in chemoinformatics, usually for predicting biological rather than physicochemical properties, naïve Bayes often being used alongside and compared against other classifiers. This has been done in studies of athletic performance enhancement,^42^ toxicity,^66^ the mechanism of phospholipidosis,^82^ and also for protein target prediction and bioactivity classification for drug-like molecules.^83^–^85^ It is in principle possible to use naïve Bayes for regression,^86^ but this is rarely seen in chemoinformatics (Table1).

**Table 1:** Some Other Machine-Learning Methods Used in Chemoinformatics

Algorithm	Description
Ant Colony^87^	Uses virtual pheromones based on ant behavior for optimization
Relevance Vector Machine (RVM)^88^	Sparse probabilistic binary classifier related to SVM; gives probabilities rather than all-or-nothing classification
Parzen-Rosenblatt Window^82^,^83^,^89^	Kernel density estimation method that allows molecular similarities to be transformed into probabilities of class membership
Fuzzy Logic^90^	Designed to give interpretable rules based on descriptor values
Rough Sets^91^	Rule-based method designed to give interpretable rules
Support Vector Inductive Logic Programming (SVILP)^84^	Rule-based method incorporating SVM ideas
Winnow^47^,^85^,^92^,^93^	For every class, Winnow learns a vector of weights for each feature. Test instances are compared with these using score thresholds
Decision Tree^23^,^76^,^94^,^95^	Like one tree from a Random Forest, but without randomization
Linear Discriminant Analysis (LDA)^96^,^97^	Models statistical differences between classes in order to make a classification
kScore^98^	Analogous to a weighted kNN scheme in which the weights are optimized by Leave-One-Out cross-validation
Projection to Latent Structures (PLS)^29^,^52^,^68^	Obtains a linear regression by projecting *x* and *y* variables to a new space. Also called Partial Least Squares

## VALIDATION

### Study Design

#### Test Sets

Chemoinformatics models are only useful if they are predictive. It is not sufficient simply to fit known data, a useful model must be able to generalize to unknown data, and thus must be validated.^99^ The traditional way of doing this is to have the total dataset divided into two parts, the *training set* and the *test set*. The training set is used to build the model, and its property values are known to the algorithm. The model is then tested on the test set, whose property values are not given to the machine-learning algorithm. However, many machine-learning approaches produce models sufficiently complex to contain internal variable parameters. Often it is helpful to optimize these parameters by holding back part of the training set as an *internal validation set*, which is used to find the parameter values giving the best predictivity.

This approach to validation is a good one, though it requires that the test set falls within the *applicability domain* of the model.^100^ This means that the training and test data should span the same region of chemical space. A test instance is unlikely to be predictable if there is no instance like it in the training set. ‘Real life’ usage of QSAR and other chemoinformatics models may well involve training and model building at one time, followed by testing on newly available data at some later date. In such cases, it is important to ensure that the new test data are within the applicability domain of the model.

#### Cross-Validation

**Figure 4:**
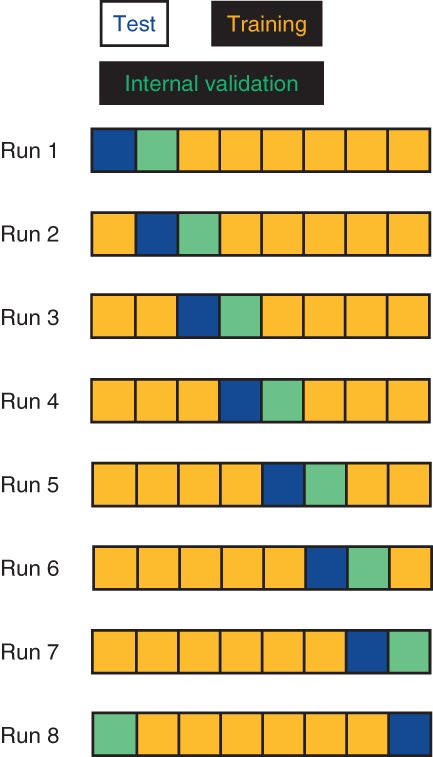
Design of a cross-validation exercise, here shown for eight-fold cross-validation. The identities of the six training, one test, and one internal validation folds are cyclically permuted.

One common and effective approach is *cross-validation*. In *n*-fold cross-validation, the data are distributed, either randomly or in a stratified way, into *n* separate folds, with one fold being the initial test set. If relevant, a second fold is used for internal validation. The remaining folds are the initial training set. The identities of the folds are then cyclically permuted, such that every fold is the test fold once—and hence each instance is predicted exactly once,^99^ see Figure 4. Thus *n* separate models are generated, and critically each instance is predicted from a model built without knowledge of that instance's property value. This requirement means that any feature selection needs to be carried out separately for each of the *n* models, ensuring that no information about the test instances finds its way directly or indirectly into the model building process. Typically, fivefold or 10-fold cross validation will be carried out. It is also fairly common to use Leave-One-Out (LOO) cross-validation, in which a separate model is built to predict each instance, trained on the remaining (*n* − 1) instances. An alternative is to use *Monte Carlo cross-validation*,^68^,^69^,^101^ in which a number of different training-test set splits are chosen randomly. Bootstrap resampling^99^ is a related approach to randomizing datasets for cross-validation, which is similar to assessing RF models by their predictivity for out-of-bag data,^39^ as discussed above. Cross-validation, Monte-Carlo or otherwise, has the advantage that it can in most study designs be repeated many times with randomly different fold definitions, with the results being averaged, leading to a more robust conclusion.

#### y-Randomization

One powerful test of a machine-learning model is *y*-randomization, also known as *y*-scrambling.^99^,^102^ The real model is compared with alternative models, which are generated from datasets in which the property values *y* are repeatedly randomly reassigned amongst the instances. The process of randomization breaks the true chemical link between the features *x* and the output property *y*, so that there is no meaningful signal left to model. If the machine learning method is still able to produce good validation statistics for the randomized models then we should be highly suspicious, as we know that it must be modeling noise rather than signal.

### Measuring Success

Measuring the success of a classification model is not as straightforward as it might initially seem. For a binary classification exercise, predictions can be classed as true positives (TP), false positives (FP), true negatives (TN), and false negatives (FN). These are combined into a *confusion matrix* of actual against predicted classes, the diagonal elements being the numbers of TP and TN, and the off-diagonal ones the numbers of FP and FN. For a multi-class classification problem, the confusion matrix is analogous to this, with correct predictions again being on the diagonal and incorrect ones off-diagonal, but it has higher dimensionality. There are numerous recipes for extracting single valued measures of prediction success from the multiple numbers in a confusion matrix, as discussed in several excellent articles.^103^–^105^ For regression models, the square of the Pearson correlation coefficient *R*^2^, together with its cross-validated counterpart *Q*^2^, are often used, as discussed by Consonni et al.^106^ Most often, the root mean squared error (RMSE) is used as the numerical measure of the prediction accuracy of a regression model, as it naturally accounts for errors of either sign. The average absolute error (AAE) is sometimes used instead and often gives substantially lower numerical values by avoiding the quadratic contributions from poorly modeled instances, as can be seen from papers such as^16^ which tabulate both measures. It is also possible to assess the number of ‘correct’ predictions if an arbitrary threshold is defined; for example, the Solubility Challenge^107^ defined any prediction of logS with an absolute error within 0.5 log_10_ units as successful.

### Interpretability

When we have identified suitable descriptors, built a chemoinformatics predictor from them, and assessed its predictive accuracy, how far can we interpret the resulting model? The model tells us that descriptor *x* is correlated with property *y*. For instance, Ploemen et al.^108^ found that induction of phospholipidosis is correlated with a molecule's acid dissociation constant pKa. Knowing that *x* can predict *y* does not tell us *how* or *why* molecules with feature *x* exhibit property *y*. There are probably several possible explanations. Maybe induction of phospholipidosis involves acid–base chemistry. Maybe it involves molecules obtaining a particular charge state, perhaps to help dissolve in an aqueous phase, or perhaps to bind to some receptor, or possibly to sequester ligands of the opposite charge? The correlation revealed by the model doesn't on its own prove one particular mechanistic hypothesis. This is what we mean when we say that QSAR, QSPR, and other chemoinformatics models ‘reveal correlation, not causation’.

So is a QSAR model useless for understanding what is going on? Clearly not. The absence of an expected correlation may allow us to reject a mechanistic hypothesis, while the presence of unanticipated correlations can suggest new hypotheses. These will need proper testing, either by direct experiment or by more sophisticated computational studies leveraging existing mechanism-relevant experimental data, as performed by Lowe et al. for phospholipidosis.^82^ So a QSAR can be a step along the road to a mechanistic understanding of a biological or chemical phenomenon, but never the final step.

Chemoinformatics models are sometimes described as ‘black boxes’. The archetypal black box would be a (virtual) machine that predicted properties excellently, but that offered no clue as to how or why they occurred. In practice, a chemoinformatics model is unlikely ever to be a completely black box. Some methods, such as multilinear regression, immediately tell us what descriptors contribute to the model; RF, at least in its implementation in R,^109^ allows this information to be extracted very easily *via* calculation of descriptor importance. Even for methods without an inherent simple measure of importance, we could (though possibly at some computational cost) build a set of models in which each descriptor is successively randomized, just as is done when a RF is ‘noised up’.^39^ Such a procedure will work best, maximizing the effect of descriptor randomization and minimizing the number of models to be built, if we first remove correlated descriptors. The fundamental point is that for any machine-learning model, even a neural network, we can examine the effect that removing input information has on the final model. Thus, we can measure and extract the importance values of descriptors from any model if we are prepared to try hard enough, as Carlsson et al. demonstrated in two cleverly designed studies that allowed them to extract chemical substructure contributions to toxicity and bioactivity from SVM and RF models.^110^,^111^

### Conclusion

Although numerous articles cited herein have compared performances of the various machine-learning algorithms used in chemoinformatics, there is no single best method for all problems. The relative abilities of methods will depend on the size and distribution in chemical space of the dataset, the linearity or otherwise of the chemical problem to be solved, the nature and internal correlation of the descriptor set available, and the relevance of nonlocal data, amongst several other factors. For linear problems, simple linear regression may be as effective as complex machine learning algorithms.^18^ For nonlocal problems, SVM and RF are probably at least as good as any other algorithm and often perform similarly when compared.^47^,^52^ The frequency with which these algorithms have been used and discussed in chemoinformatics, together with their easy availability *via* platforms such as R,^109^ makes SVM and RF good starting points for chemists beginning to incorporate machine learning into their work. For problems where only local data are likely to be relevant, kNN is an excellent and simple approach.^69^,^70^

Validation is seen to be a critical part of any machine-learning project and the design of the *in silico* experiment is crucial to the robustness of the study. A traditional training-test split of the data is a good validation strategy, provided that the two sets span the same regions of chemical space. Cross-validation is also a popular strategy, and still allows models to be tested on data unseen in their generation. *y*-randomization provides a useful test of a chemoinformatics model; a predictor that can find a signal in random data is not to be trusted. Various different metrics are used for measuring success, with RMSE and *R*^2^ typically being used for regression studies. Several different metrics for binary and multi-class classification exist, all being derived from the confusion matrix. QSAR and QSPR models reveal correlation between descriptors and properties, but do not by themselves prove mechanistic hypotheses.
